# The Expansion of Thymopoiesis in Neonatal Mice Is Dependent on Expression of *High Mobility Group A 2 Protein (Hmga2)*

**DOI:** 10.1371/journal.pone.0125414

**Published:** 2015-05-01

**Authors:** Beata Berent-Maoz, Encarnacion Montecino-Rodriguez, Michael Fice, David Casero, Christopher S. Seet, Gay M. Crooks, William Lowry, Kenneth Dorshkind

**Affiliations:** 1 Department of Pathology and Laboratory Medicine, David Geffen School of Medicine at UCLA, University of California Los Angeles, Los Angeles, California, United States of America; 2 Department of Medicine, Division of Hematology-Oncology, David Geffen School of Medicine at UCLA, University of California Los Angeles, Los Angeles, California, United States of America; 3 Department of Pediatrics, David Geffen School of Medicine at UCLA, University of California Los Angeles, Los Angeles, California, United States of America; 4 Department of Molecular, Cell and Developmental Biology, University of California Los Angeles, Los Angeles, California, United States of America; Oklahoma Medical Research Foundation, UNITED STATES

## Abstract

Cell number in the mouse thymus increases steadily during the first two weeks after birth. It then plateaus and begins to decline by seven weeks after birth. The factors governing these dramatic changes in cell production are not well understood. The data herein correlate levels of *High mobility group A 2* protein (*Hmga2*) expression with these temporal changes in thymopoiesis. *Hmga2* is expressed at high levels in murine fetal and neonatal early T cell progenitors (ETP), which are the most immature intrathymic precursors, and becomes almost undetectable in these progenitors after 5 weeks of age. *Hmga2* expression is critical for the initial, exponential expansion of thymopoiesis, as *Hmga2* deficient mice have a deficit of ETPs within days after birth, and total thymocyte number is repressed compared to wild type littermates. Finally, our data raise the possibility that similar events occur in humans, because *Hmga2* expression is high in human fetal thymic progenitors and falls in these cells during early infancy.

## Introduction

The rate of T cell production in the murine thymus varies considerably at different stages of life. One of the most dramatic fluctuations occurs between fetal and young adult life. The key feature of this stage of development is the increase in thymus cellularity that is critical for generating the high number of thymocytes that colonize secondary lymphoid tissues and establishing the T cell repertoire. Thymus cell number increases exponentially through the first two weeks after birth. It then plateaus, and thymopoiesis begins to decline by seven weeks after birth [1, 2]. The factors governing these dramatic shifts in cell production are not well understood.

The most immature progenitors in the murine thymus are early T lineage progenitors (ETP), and their progeny include double negative (DN) 2, DN3, and DN4 thymocytes [3]. The latter cells are the precursors of more mature thymocytes that ultimately leave the thymus and colonize peripheral lymphoid tissues. As part of our efforts to define age-related changes in ETP, we harvested them from mice of different ages and performed whole transcriptome profiling. This analysis revealed major differences in patterns of gene expression between young and old ETP, and we were particularly struck by the significantly reduced expression of the gene encoding *high mobility group A 2* protein (*Hmga2)*.

*Hmga2* is expressed most robustly in fetal hematopoietic stem cells (HSCs) and is down-regulated within weeks after birth [4]. This change in *Hmga2* expression results in a reduction of HSC self-renewal and regenerative potential [4, 5], which in turn contributes to the switch from highly active fetal to steady-state adult hematopoiesis that occurs in mice by six weeks after birth [6–8]. In view of these effects in HSCs, we questioned whether changes in *Hmga2* expression might also be involved in the dramatic fluctuations in thymus cell production occurring in neonatal and young adult mice.

We now report that *Hmga2* is expressed at high levels in ETPs from fetal and neonatal mice and that levels fall significantly in these progenitors after five weeks of age. We also demonstrate that *Hmga2* deficient mice have a severe ETP deficit and that neonatal thymopoiesis in that strain is severely depressed. Together, these results implicate changes in *Hmga2* expression in the initial expansion and decline of thymopoiesis that occurs in the neonate and young adult, respectively, and suggest that these fluctuations in cell production reflect the transition from fetal to adult hematopoiesis. Finally, we demonstrate that *Hmga2* is expressed in fetal, but not infant, ETPs, suggesting that HMGA2 regulates early transitions during human thymopoiesis.

## Materials and Methods

### Mice

Fetal, neonatal, and young adult C57BL/6J (B6) mice were obtained from the UCLA Division of Laboratory Animal Medicine. Seventy-two week old B6 mice were purchased from the National Institute on Aging colony. Timed pregnant C57BL/6J mice were purchased from Jackson Laboratories or produced in the UCLA Division of Laboratory Animal Medicine. *Hmga2* ^-/-^ mice and their normal controls were obtained from Drs. William Lowry and Andrew White at UCLA and were generated as previously described [9]. In some cases, *Hmga2* ^-/-^ mice carried Lox-Stop-Lox KrasG12D, Lox-Stop-Lox YFP and two p53 floxed alleles. Since none of the examined animals carried a Cre allele, KrasG12D and YFP were never expressed and p53 was never deleted. Because *Hmga2* ^-/-^ mice are smaller than their normal counterparts, data are presented as a indexes relative to weight (total thymocyte number/animal weight in grams) as previously described [10]. Animals were housed in the Division of Laboratory Animal Medicine and sacrificed by CO_2_ inhalation as per institutional animal care guidelines.

### Human thymus

Human fetal thymic tissue was obtained from Novogenix (Los Angeles, CA). Human infant thymii were obtained from healthy donors via the Translational Pathology Core Laboratory housed in the Department of Pathology and Laboratory Medicine and the Jonsson Comprehensive Cancer Center. Anonymized tissues were obtained according to guidelines approved by the Institutional Review Board of the University of California, Los Angeles.

### Whole transcriptome sequencing

Following isolation of RNA, cDNA libraries were built using the Nugen Ovation RNA-Seq System v2 and Illumina’s TrueSeq DNA Sample Preparation kit V2 (FC-121-2001). An Agilent Bioanalyzer was used to determine RNA quality prior to sequencing. RNA-Seq libraries were built and sequenced in the Jonsson Comprehensive Cancer Center’s Gene Expression core laboratory. Libraries were sequenced on an Illumina HiSeq 2000 (paired-end 100bp). Raw sequence files were obtained using Illumina’s proprietary software and are available at NCBI’s Gene Expression Omnibus (Accession Number: GSE67112). RNA-seq reads were aligned using STAR v2.3.0 [11]. The GRCm38 assembly (mm10) of the mouse genome and the junction database from Ensemble’s gene annotation (release 71) were used as reference for STAR. The count matrix for genes in the UCSC genome annotation was generated with HTSeq-count v0.5.4p3 [12] and normalized using the geometric mean across samples [13]. Both cuffdiff v2.1.1 [14] and DESeq v1.14.0 [13] were used to classify genes as differentially expressed (Benjamini-Hochberg adjusted p-value < 0.05). Moderate fold changes between conditions were obtained from variance-stabilized data [13]. Functional annotation of differentially expressed genes was generated through the use of IPA (Ingenuity Systems, www.ingenuity.com). Hierarchical gene and functional clustering was performed with GENE-E (http://www.broadinstitute.org/cancer/software/GENE-E/).

### Immunofluoresence and flow cytometry

**Fig 1:**
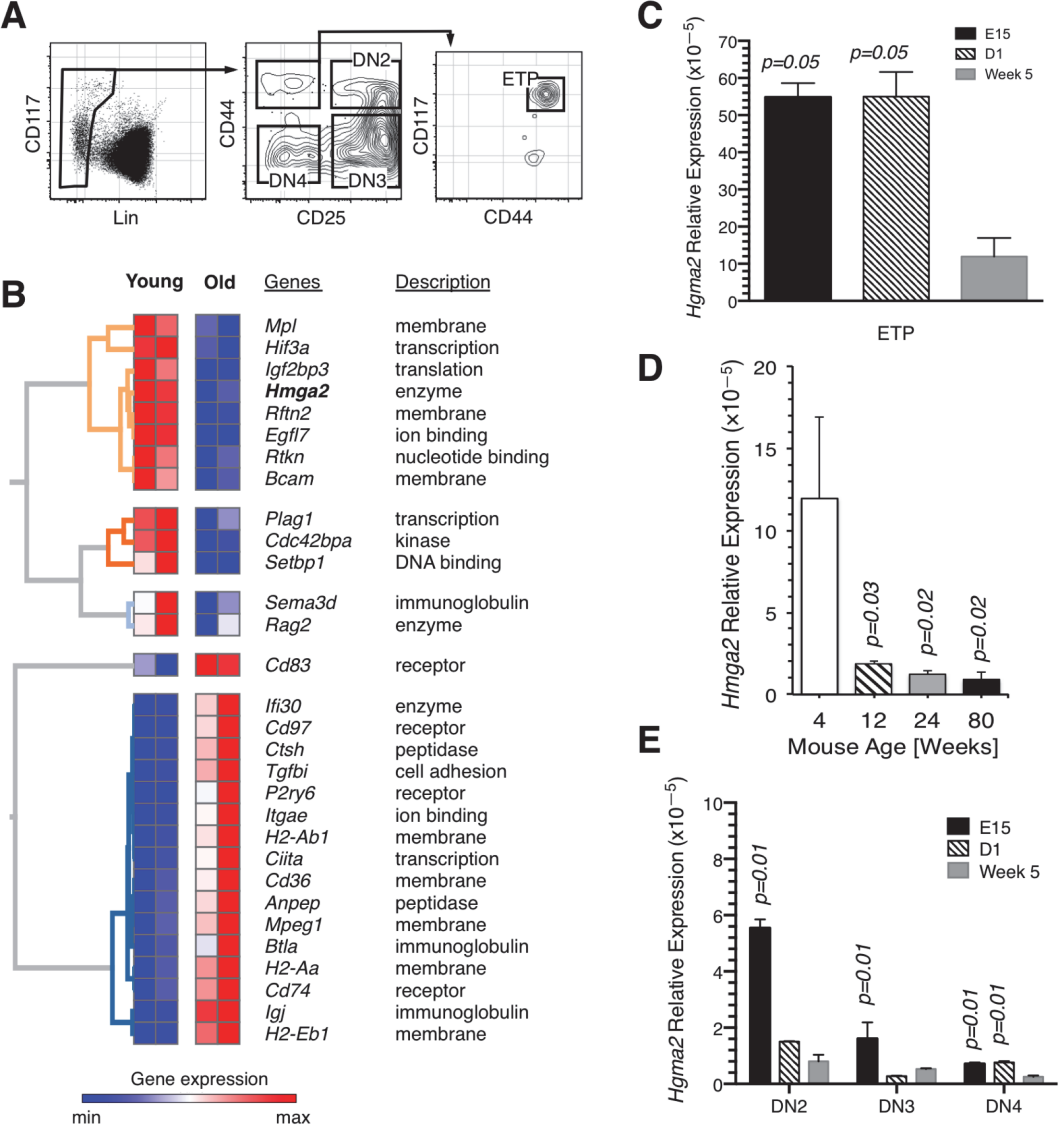
Whole transcriptome analysis of ETP. (A) Resolution of ETP and double negative thymocyte subsets. Live cells were gated based on lineage negativity (Lin^-^) and CD44 and CD25 levels of expression as shown. ETP were further resolved as Lin^-^ CD44^+^ CD25^-^ CD117^+^ cells. The thymus of 6 week old B6 mice was used to obtain the data shown in the FACS plots. (B) Hierarchical clustering of selected genes. Variance stabilized RNA-seq expression estimates are shown for each gene and replicated condition. The color scale shows the relative value. Gene descriptions are from Gene Ontology molecular function annotations. (C) Expression of *Hmga2* relative to *Gapdh* in ETP from E15 embryos (E15), day 1 neonates (D1), and 5 week old (Week 5) B6 mice measured by qPCR. (D) Expression of *Hmga2* relative to *Gapdh* in ETP harvested from 4 week, 12 week, 24 week, and 80 week old B6 mice measured by qPCR. (E) Expression of *Hmga2* relative to *Gapdh* in DN2, DN3 and DN4 progenitors isolated from E15 embryos (E15), day 1 neonates (D1), and 5 week old (Week 5) B6 mice measured by qPCR. The data in panels (C) and (E) are from the same group of mice.

Murine ETP and double negative (DN) thymocyte subpopulations were resolved as previously described in detail [15] and the gating strategy used is shown in Fig 1A. Thymocyte cell suspensions were prepared and cells were incubated with anti-CD16/CD32 (FcγRII-III; clone 2.4G2, eBiosciences) as a blocking agent and a cocktail of anti-lineage monoclonal antibodies that included goat-anti-mouse IgM (Southern Biotechnology), CD3ε (145-2C11), CD8α (53–6.7), NK-1.1 (PK136), TCRβ (H57-597), TCRγδ (UC7-13D5), Gr-1 (RB6-8C5), CD11b (M1/70), CD45R(B220; RA3-6B2), TER-119 (TER119), CD117 (c-kit, clone 2B8), CD25 (clone PC61.5) (all from eBiosciences) and CD44 (clone IM7, BD Biosciences). Antibodies to CD117, CD25 (clone PC61.5; eBiosciences) and CD44 (clone IM7, BD Biosciences) were used to define ETP and DN2, DN3 and DN4 populations in combination with the above lineage cocktail. Thymocyte subpopulations were analyzed on an LSR II (Becton-Dickinson) or sorted using a FACSaria flow cytometer (Becton Dickinson) located in the Broad Stem Cell Research Center and the Jonsson Cancer Center Flow Cytometry Core at UCLA, respectively.

Human T cell progenitors were isolated as follows: Single-cell suspensions were prepared from thymic tissue by fine dissection in serum-free RPMI. Mononuclear cells were isolated by density gradient centrifugation over Ficoll-Paque Plus (GE Healthcare) and enriched for CD34^+^ cells by positive selection using MACS magnetic beads and LS separation columns (Miltenyi Biotech). The CD34^+^ and CD34^-^ fractions were incubated with the following mouse anti-human monoclonal antibodies: FITC-labeled lineage-specific antibodies, which included anti-CD3 (clone UCHT1), CD14 (clone M5E2), CD15 (clone HI98), CD19 (HIB19), CD56 (clone B159), and CD235a (glycophorin A, clone GA-R2), all from BD Biosciences. Additional markers used for progenitor isolation were Brilliant Violet 421 CD45 (clone HI30), APC-Cy7 CD34 (clone 581), and APC CD1a (clone HI149), all from Biolegend. Live cells were discriminated by DAPI exclusion, and human T cell progenitors were resolved and FACS purified based on their lineage negative (Lin^—^) CD45^+^ CD34^+^ CD1a^–^, Lin^—^ CD45^+^ CD34^+^ CD1a^+^, and Lin^—^ CD45^+^ CD34^—^ phenotypes.

### qPCR

RNA was extracted with the RNeasy Plus micro- or minikit and cDNA was synthesized with RT^2^ First Strand kit (both from QIAGEN) or TaqMan MicroRNA Reverse Transcription Kit (Applied Biosystems). PCR reactions were run using SYBR green qPCR master mix (Bio-Rad, Hercules, CA) or TaqMan Universal PCR master mix (Applied Biosystems) as per manufacturer instructions. Data were analyzed with Bio-Rad IQ5 software using the *Pfaffl* method. The reference genes are indicated in the figure legends. Amplification efficiencies were routinely found to be between 95% and 105%. All reactions were run at least twice in duplicate. *Ink4a* primer sequences were as follows: GTGTGCATGACGTGCGGG (forward) and GCAGTTCGAATCTGCACCGTAG (reverse). TaqMan gene expression assays for mouse *Hmga2*, *Lin28b*, *β-Actin*, *Gapdh* and human *Hmga2*, *Lin28b*, *β-2-microglobulin* as well as TaqMan MicroRNA Assays for *Sno202* and *Let7b* were purchased from Applied Biosystems.

### Statistical analysis

Data are expressed as a mean ± SD or SEM as indicated in the figure legends. Differences between groups were tested by a two-tailed, unpaired Student’s t test (α = 0.05).

### Study approval

Animal experiments were conducted according to UCLA Institutional Animal Care and Use Committee guidelines (IACUC). The mouse experiments described herein were approved by the IACUC in protocol ARC #1996-164-61 (approval period from 4/11/2014 through 4/10/2017). De-identified human fetal thymic samples were obtained from Novogenix (Los Angeles, CA) following informed consent performed in accordance with federal regulations (Subpart B: 45 CFR 46.206). Human post-natal thymic samples were obtained as waste tissue from patients during cardiothoracic surgery and provided without patient identifiers according to guidelines approved by the UCLA Institutional Review Board (IRB). According to UCLA policy, investigators who use completely anonymized human tissue samples do not need IRB approval. Details regarding this policy can be found on the UCLA Translational Pathology Core Laboratory web site (http://pathology.ucla.edu/body.cfm?id=133).

## Results and Discussion

As part of our studies of how aging affects ETP, we compared patterns of gene expression in young and old murine progenitors, isolated as shown in Fig 1A, by whole transcriptome profiling. We observed that there were significant differences in the expression of genes that regulate key cellular processes between young and old progenitors, including differentiation, survival, DNA repair, migration, and proliferation. A sampling of genes whose expression differed in ETP based on developmental age is shown in Fig 1B, and this included *Hmga2*, which was down-regulated approximately thirty-fold between the young and old cells.

Because reductions in *Hmga2* expression in HSCs are evident in young adult mice [4], we refocused our studies from aging per se to determine the precise time at which declines occur in ETP. We harvested these progenitors from fetal, newborn, young adult, and old mice and quantified *Hmga2* levels using quantitative PCR (qPCR). These analyses demonstrated that there is a decline in *Hmga2* expression in ETP following the neonatal period. As shown in Fig 1C and 1D, ETP from five week-old mice expressed *Hmga2* at levels that were five-fold lower than in fetal and neonatal progenitors, and levels had declined even further by twelve weeks of age. In retrospect, we were fortunate that our young age group that was transcriptionally profiled included four week old mice; if our young cohort had been a few weeks older, we would likely have failed to detect any declines in *Hmga2* expression compared to the old progenitors.

The above results indicate that in addition to HSCs [4], *Hmga2* is expressed in T lineage specified progenitors. The various stages of murine thymopoiesis are well defined, providing the opportunity to determine whether *Hmga2* is expressed at all stages of T cell development or it is confined to the most immature progenitors. We isolated double negative (DN) 2, DN3, and DN4 progenitor populations (Fig 1A), and *Hmga2* expression was examined by qPCR.

In general, overall levels of expression were ten to thirty times lower in DN2-4 progenitors compared to those in ETP regardless of age. However, consistent with what was observed in ETP, there was a significant decline in *Hmga2* expression in the DN subsets by 5 weeks after birth (Fig 1E). Together, these data suggest that in the mouse thymus, *Hmga2* expression is most prominent in the most immature T lineage specified progenitors.

The high expression of *Hmga2* in fetal/neonatal ETP and the declining levels after five weeks of age correlate quite well with what is known about the kinetics of cell production in the murine thymus. In this regard, there is a continual increase in the number of thymocytes through the first two weeks after birth. Cell production then plateaus, and total organ cellularity begins to decline by seven weeks after birth [1, 2]. Because these changes in T cell production parallel the pattern of *Hmga2* expression, we considered the possibility that HMGA2 regulates the size of the ETP compartment and cell production in the newborn thymus. We examined thymopoiesis in fetal, neonatal, and young adult *Hmga2* ^*-/-*^ mice in order to assess this possibility.

**Fig 2:**
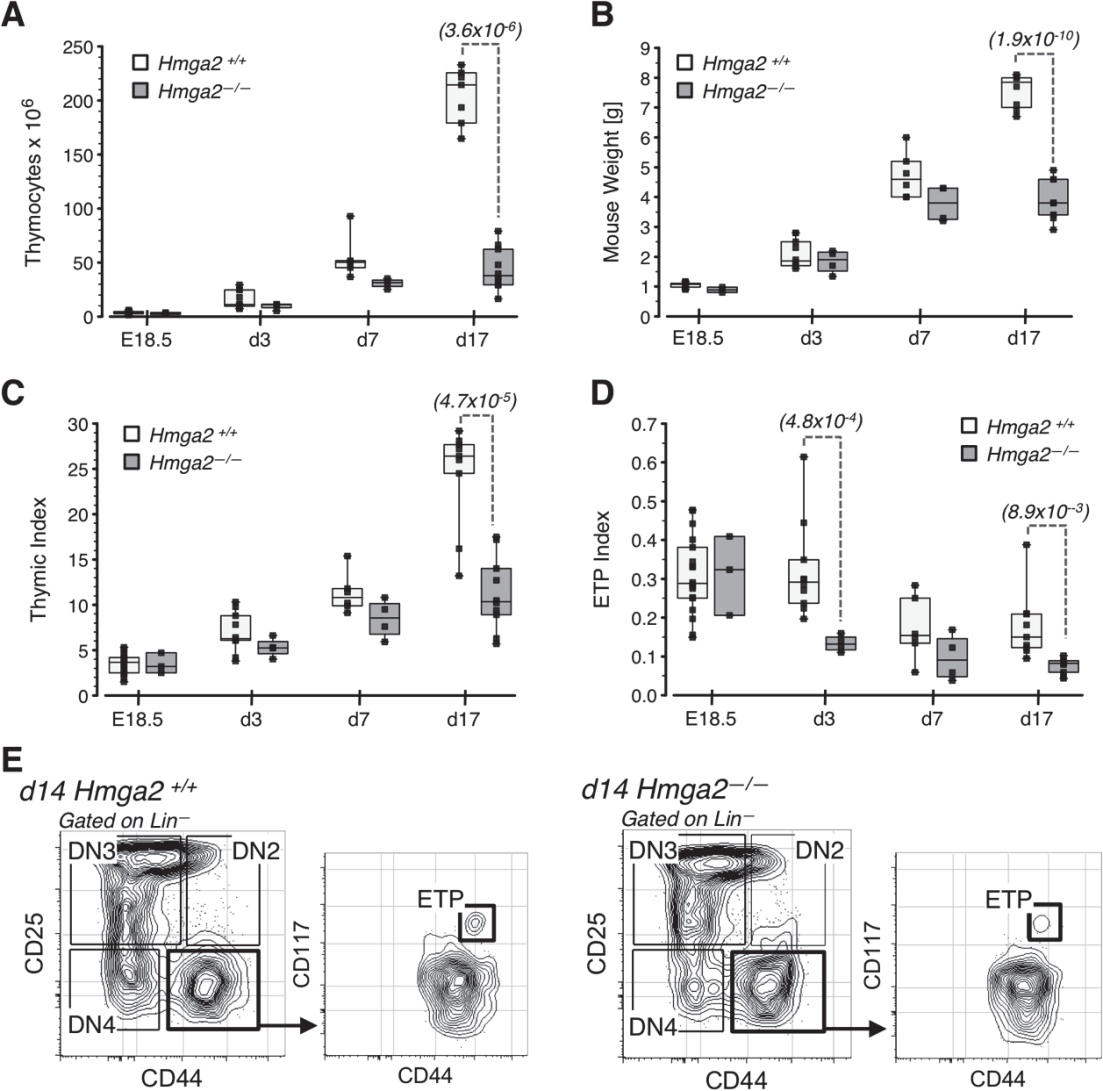
Thymopoiesis in *Hmga2* ^-/-^ mice. (A) Total thymocyte numbers, (B) Mouse weight in grams, and (C) Thymic Index (thymocyte number/mouse weight in grams) for *Hmga2* ^*-/-*^ and *Hmga2* ^*+/+*^ mice of the indicated ages. (D) ETP Index, defined as ETP number/mouse weight in grams for each mouse. The boxes and lines in panels A-D indicate the 95% confidence interval and median for each group, respectively. Each symbol represents a mouse. The number of animals per group were as follows: *Hmga2* ^*-/-*^ (E18.5, n = 3; d3, n = 4; d7, n = 2, and d17, n = 5) and *Hmga2* ^*+/+*^ (E18.5, n = 18; d3; *n = 10; d7*,*n = 2*, *and d17*, *n = 6)*. The indices were used in order to account for differences in size and weight between *Hmga2* ^*-/-*^ and *Hmga2* ^*+/+*^ mice. (E) Representative FACS plots of ETP from day 14 *Hmga2* ^-/-^ and *Hmga2* ^*+/+*^ mice.

Thymic cellularity was similar between fetal *Hmga2* ^*-/-*^ and *Hmga2* ^+/+^ mice as well as neonates through the first week after birth. However, by day 17 after birth thymocyte numbers were significantly lower in *Hmga2* ^*-/-*^ compared to *Hmga2* ^+/+^ mice (Fig 2A). As *Hmga2* ^-/-^ mice develop, they are noticeably smaller than their wild type littermates (Fig 2B). In order to take this into account when comparing knockout and wild type mice, a cellularity index (thymocyte number/animal weight in grams) was calculated as previously described [10]. This data normalization confirmed that thymopoiesis was similar in *Hmga2* ^*-/-*^ and *Hmga2* ^+/+^ mice up until the first week after birth but was reduced thereafter (Fig 2C).

A similar approach was used to quantify ETP. There was no difference in the fetal ETP index, indicating that *Hmga2* expression is not required for the initial development of ETPs. However, the *Hmga2* ^-/-^ ETP index was significantly lower compared to that in wild type littermates by three days after birth (Fig 2D), and this decline preceded the decrease in total organ cellularity. The fact that the frequency of ETPs is reduced in the thymus of 2 week old *Hmga2* ^-/-^ mice is consistent with these results (Fig 2E).

It is unlikely that the initial declines in ETP number are due to their diminished production from bone marrow precursors, because the number of HSCs in fetal *Hmga2* ^-/-^ and *Hmga2* ^+/+^ mice is similar [4]. Instead, intrinsic defects in ETP that result from HMGA2 deficiency are likely operative. These may include the same deficiencies in self-renewal that have been ascribed to *Hmga2* deficient HSCs [4]. Further studies are needed to assess how *Hmga2* expression affects this property. Unfortunately, unlike HSCs whose self-renewal potential can be measured using in vivo transplantation assays, the limited self-renewal potential of even wild type ETPs precludes the use of similar in vivo approaches.

Declines in thymopoiesis initiate within the first year of life in humans [16]. The volume of the thymic epithelial space is reduced within a year of birth [17], and there is a decrease in the total number of cells that express T cell receptor excision circles, which are used to estimate the levels of newly produced thymocytes [18]. In view of this, we questioned whether *Hmga2* expression patterns in human thymocyte progenitors also differed according to age as we observed in mice.

**Fig 3:**
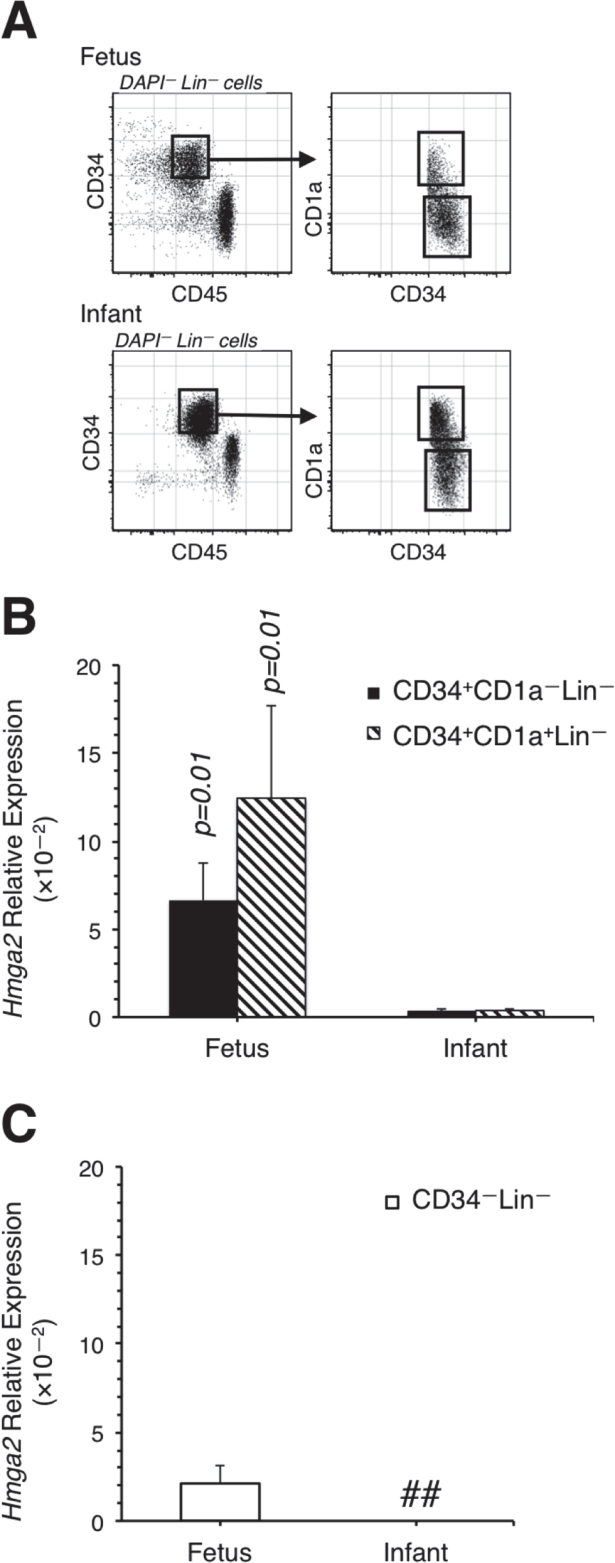
*Hmga2* expression is higher in human fetal versus infant thymic progenitors. (A) FACS plots showing the resolution of human lineage negative T cell progenitors according to CD34 and CD1a expression. The plots are representative of two independent sorts for each sample. (B) Expression of *Hmga2* in Lin^-^ CD34^+^ CD1a^-^ and Lin^-^ CD34^+^ CD1a^+^ T cell progenitors isolated from 15.5 weeks gestation fetus (Estimated Developmental Age) and 6 month old human (infant) thymii. (C) Expression of *Hmga2* in Lin^-^ CD34^-^ fetal and infant thymocytes. T cell subsets were isolated from two independent samples of each age. Data represent mean ± SD. All reactions were run twice.

We isolated T cell progenitors from the thymus of two 15 week old fetuses and two 6 month old infants. The lineage negative (Lin^–^) CD34^+^ CD1a^–^ cells are the equivalent of murine ETP while the Lin^—^ CD34^+^ CD1a^+^ pre-T cells include early DN subsets (Fig 3A) [19]. We also purified Lin^—^ CD34^—^ thymocytes which would include later DN cells. The data show that, similar to what was observed in murine ETP, human ETPs and early DN cells from the fetal thymus expressed high levels of *Hmga2* while expression was nearly undetectable in progenitors from 6 month old infants (Fig 3B and 3C). The data also show that, as in mice, more mature DN cells express relatively low levels of *Hmga2* (Fig 3C).

**Fig 4:**
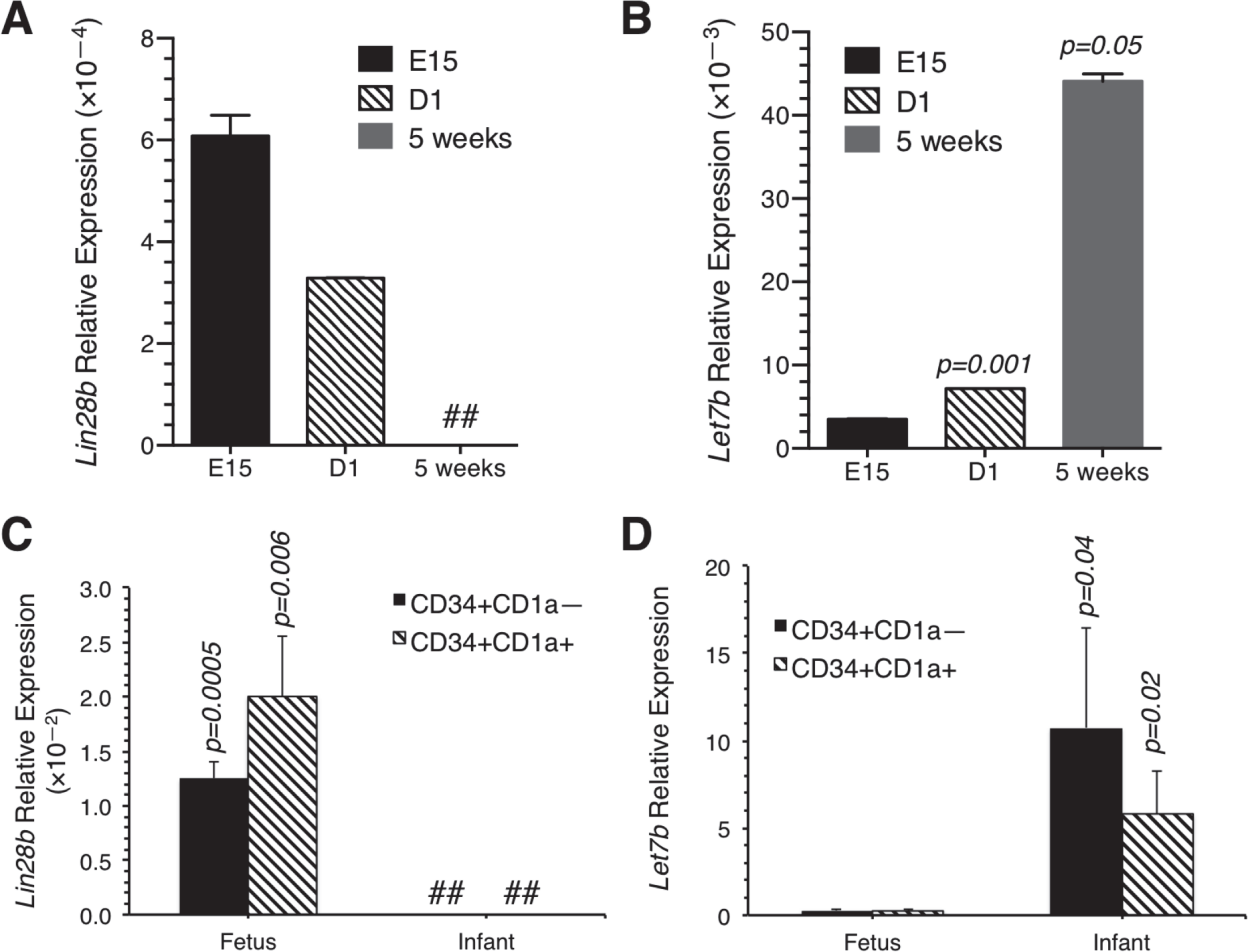
Expression of *Lin28b* and *let7b* in murine and human thymic progenitors. (A) Expression of *Lin28b* relative to *Gapdh* in ETP from E15 embryos (E15), day 1 neonates (D1), and 5 week old (5 weeks) mice by qPCR. *Lin28b* expression was undetectable in ETP from 5 week old mice. Data shown represent means ± SD. (B) Expression of *Let7b* relative to *Sno202* in ETP, DN2, DN3 and DN4 progenitors from E15, D1, and 5 week old B6 mice. Levels of *let7b* are significantly higher in ETP from D1 and 5 week old mice compared to cells from E15 embryos. Data shown represent means ± SD. In A and B, wild type thymii from 6–10 fetuses and 6–8 day 1 neonates were pooled and ETP isolated. The 5 week old mice (4–6 animals) were processed individually. qPCR reactions were run twice. (C) Expression of *Lin28b* in human Lin^-^ CD34^+^ CD1a^-^ and Lin^-^ CD34^+^ CD1a^+^ T cell progenitors. Expression was higher in the fetal progenitors. (D) Expression of *let7b* relative to *Sno202* in human Lin^-^ CD34^+^ CD1a^-^ and Lin^-^ CD34^+^ CDa1^+^ T cell progenitors. Expression was significantly higher in the infant compared to fetal progenitors. ##; not detected. p values are indicated on the figure.

*Hmga2* expression in murine HSCs is regulated by the let7 microRNA which inhibits *Hmga2* mRNA translation and *let7b* processing is in turn blocked by Lin28b [4]. Similar to what occurs in HSCs, ETP from fetal mice expressed high *Lin28b* levels that declined by one day after birth, and expression was undetectable in progenitors harvested from 5 week old animals (Fig 4A). The age-related decline in *Lin28b* inversely correlated with *let7b* expression, with low *let7b* in fetal ETP and relatively high levels in ETP from 5 week old mice (Fig 4B). Further similarities between mice and humans were observed when *Lin28b* and *let7b* expression levels were examined in fetal and infant thymic progenitors. Human Lin^—^ CD34^+^ CD1a^—^ and Lin—CD34^+^ CD1a^+^ fetal progenitors expressed high levels of *Lin28b* while *Lin28b* was undetectable in cells from 6 month old infants (Fig 4C). Conversely, *let7b* levels were almost undetectable in fetal T cell progenitors while expression was significantly increased in 6 month old thymocytes (Fig 4D). These results are consistent with a recent report that *Lin28b* is expressed in human fetal hematopoietic tissues but not adult bone marrow [20].

## Conclusions

The number of cells in the mouse thymus increases exponentially through the first two weeks after birth [1, 2]. Our data demonstrate that this initial growth phase is dependent at least partially on *Hmga2* expression, because thymocyte expansion does not occur in *Hmga2* deficient mice. Our expression data also suggest a role for HMGA2 during early human thymopoiesis. The initial expansion of thymopoiesis does not occur in *Hmga2* deficient mice. The thymopoietic defects in that strain could result from lack of *Hmga2* expression in multiple intrathymic and extrathymic compartments. However, our data showing expression of *Hmga2* in ETP indicate that these include effects manifest in that compartment.

**Fig 5:**
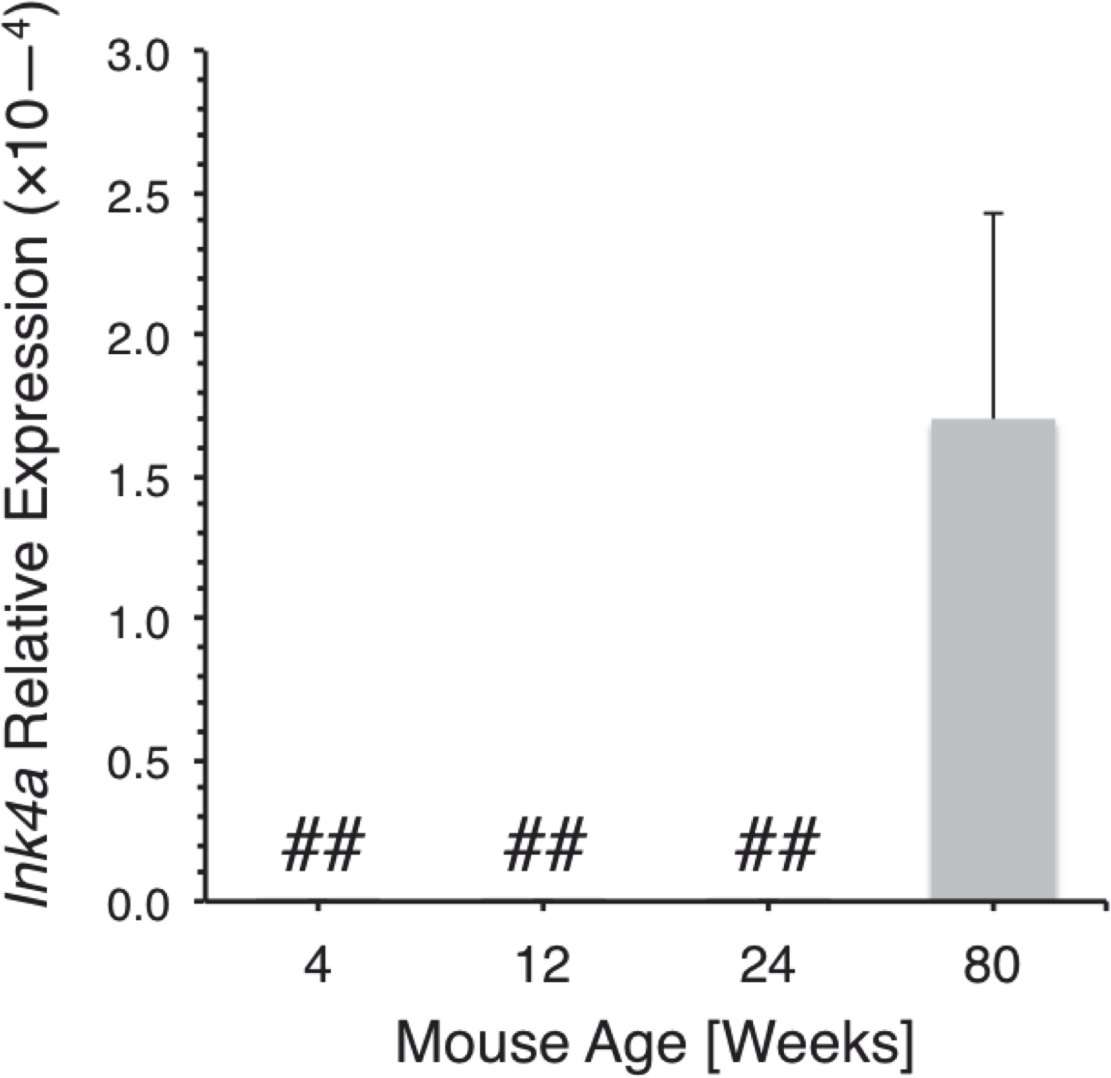
Ink4a is not expressed in neonatal ETP. *Ink4a* expression as measured by qPCR relative to *Gapdh* in ETP purified from the thymus of two 4 week old, three 12 week old, three 24 week old, and eight 80 week old mice. Data represent mean ± SD. Thymii from the various aged mice were pooled and ETP were isolated. All reactions were run twice on each sample.

We also correlate the subsequent decline in thymocyte number with the repression of *Hmga2* expression. These changes in the tempo of thymopoiesis are likely not due to the aging process per se, because they occur within weeks after birth and are not associated with ETP expression of senescence associated genes such as *Ink4a* [21]. In this regard, Ink4a expression in ETP is not detected within the first six months after birth (Fig 5).

**Fig 6:**
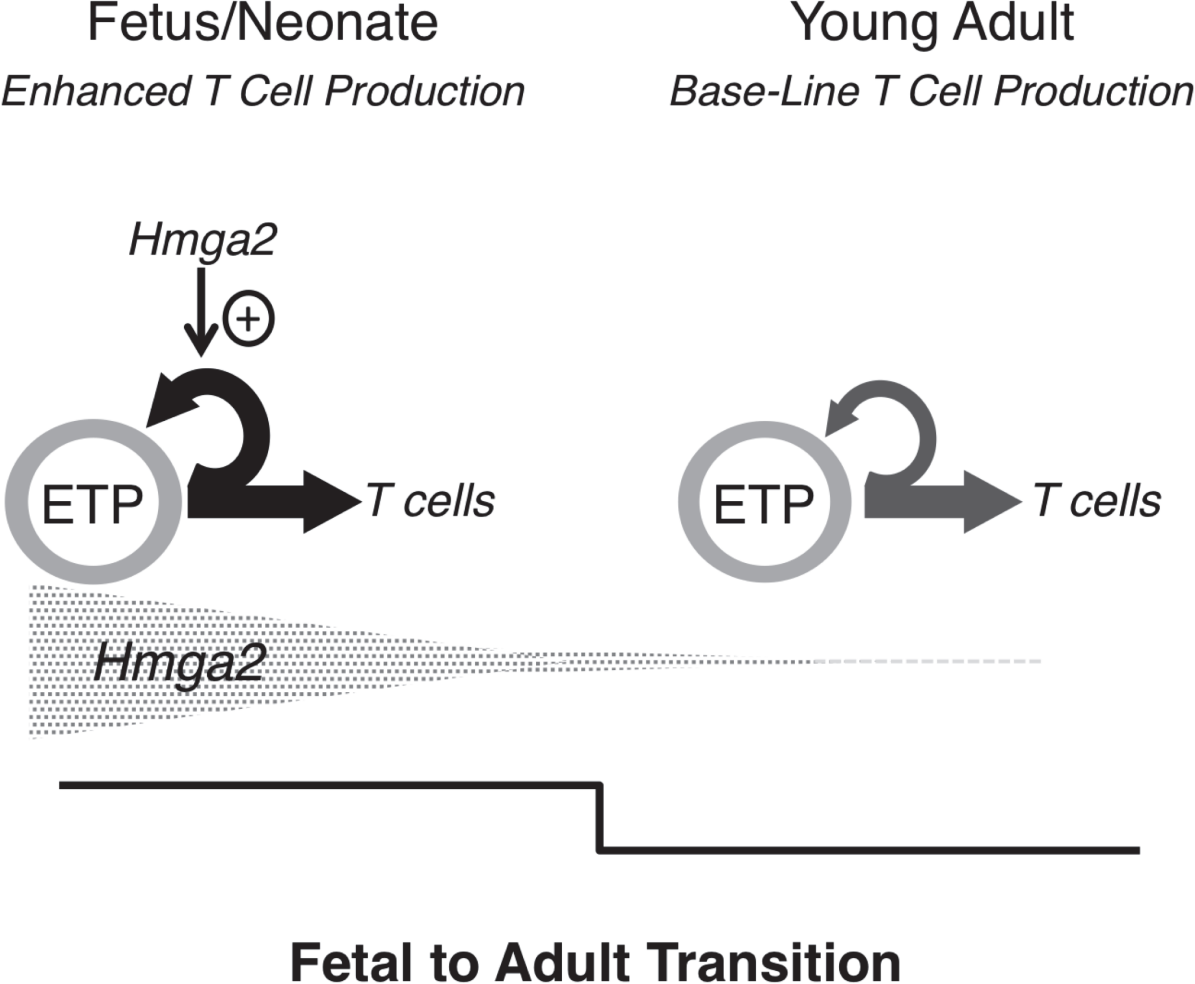
Model of how *Hmga2* expression affects cell production in the fetal and young adult thymus. During the fetal/neonatal phase of thymopoiesis, high levels of *Hmga2* contribute to efficient maintenance of the ETP compartment, possibly through enhanced proliferation and/or self-renewal. This in turn contributes to the high rate of cell production in the fetal/neonatal thymus. During the transition from the fetus/neonate to the young adult, there is a sharp down-regulation of *Hmga2* expression in ETP which contributes to the diminution in the tempo of thymopoiesis to steady state adult levels.

Instead, the data suggest that the changes in the pattern of T cell production in the thymus between neonatal and young adult life reflect the switch from highly active fetal to steady state adult hematopoiesis (Fig 6).
